# Supplementary material for: Musculoskeletal Injury Risk in a Military Cadet Population Participating in an Injury-Prevention Program
Source: Medicina (Kaunas). 2023 Feb 13;59(2):356. doi: 10.3390/medicina59020356 (PMC9961050; doi:10.3390/medicina59020356)

INJURY-PREVENTION PROGRAM

Part 1. Range of motion and bodyweight strength exercises. 10 min

Neck

- neck lateral flexion, 5x bilaterally

Controlled, smooth movement within comfortable limits.

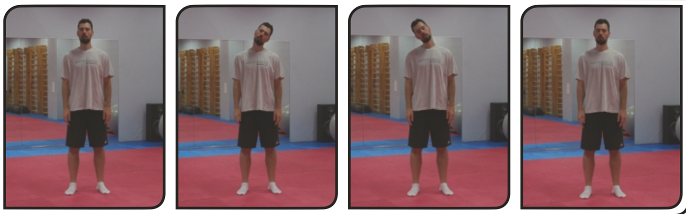

- neck rotation, 5x bilaterally

Controlled, smooth movement within comfortable limits.

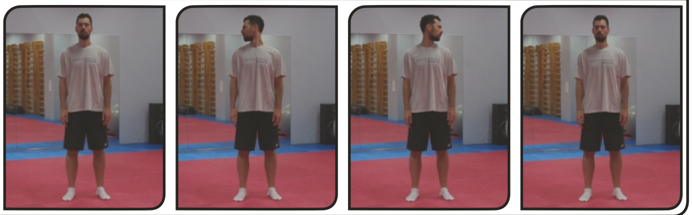

Shoulder

- shoulder flexion-extension, 5x each

Controlled, smooth movement within comfortable limits.
During movement, palms facing each other.

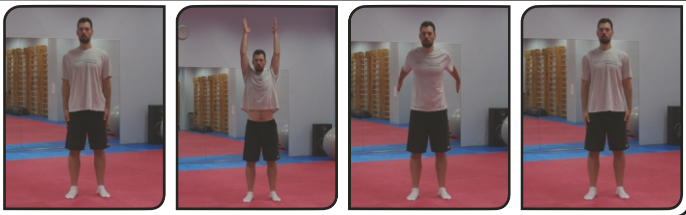

- shoulder abduction-adduction, 5x each

Controlled, smooth movement within comfortable limits.
During movement, palms facing forward.

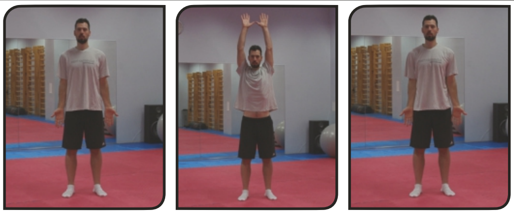

- shoulder internal-external rotation, 5x each

Controlled, smooth movement within comfortable limits.
During rotation, 90 degree shoulder abduction and elbow flexion.

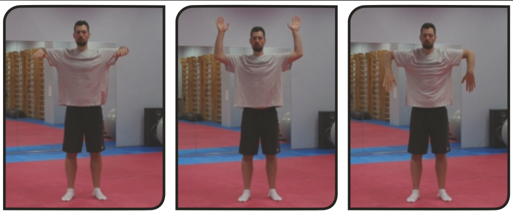

- shoulder Y raise, 10x

Controlled, smooth movement within comfortable limits.

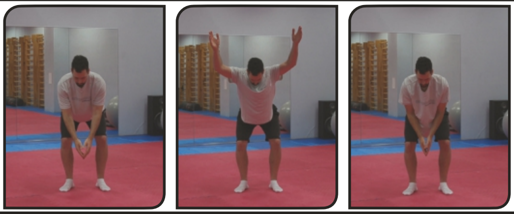

- push-up plus, 10x

Controlled, smooth movement within comfortable limits.

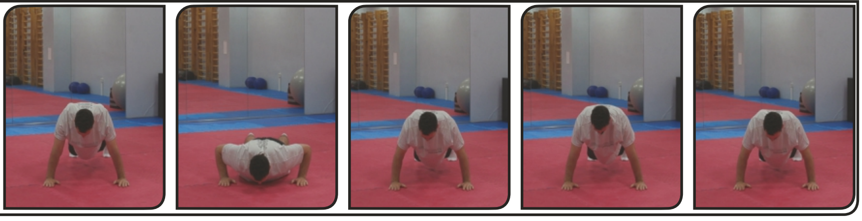

Trunk

- hip hinge squat-trunk extension, 5x

Controlled, smooth movement within comfortable limits.
While squatting, maintain slight extension in the lower back.

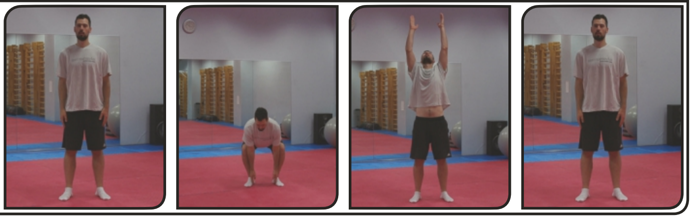

- trunk lateral flexion, 5x bilaterally

Controlled, smooth movement within comfortable limits.

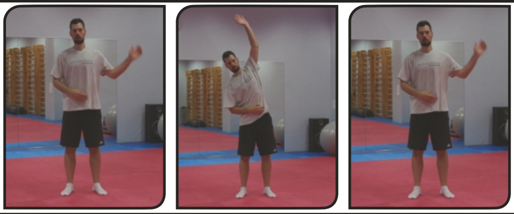

- trunk rotation, 5x bilaterally

Controlled, smooth movement within comfortable limits.

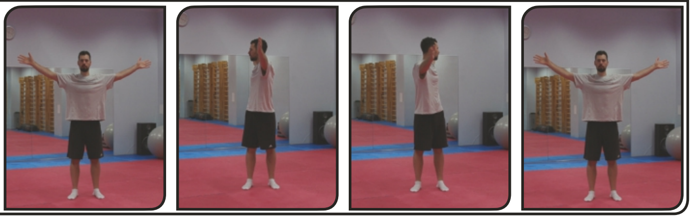

- knee to opposite elbow, 5x each

Controlled, smooth movement within comfortable limits.
In the push-up start position, bring your knee, with trunk rotation and hip flexion, close to the opposite elbow.

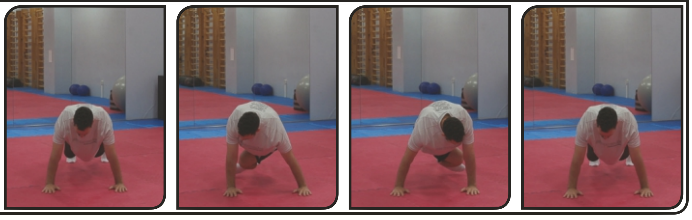

- prone bridge trunk rotation, 5x bilaterally

Controlled, smooth movement within comfortable limits.

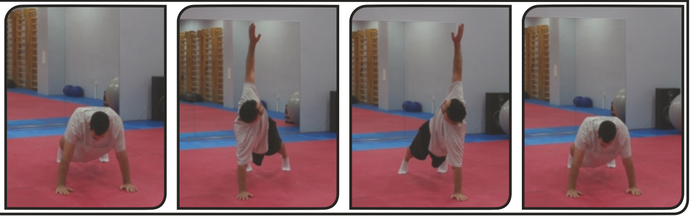

Hip/knee

- hip and knee flexion, 5x bilaterally

Controlled, smooth movement within comfortable limits.

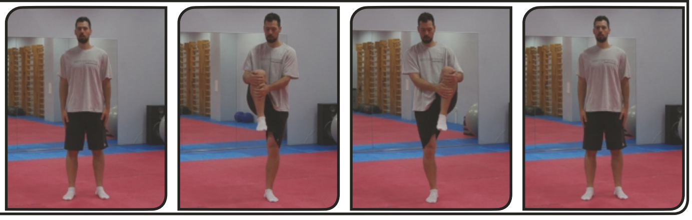

- knee flexion, 5x each

Controlled, smooth movement within comfortable limits.

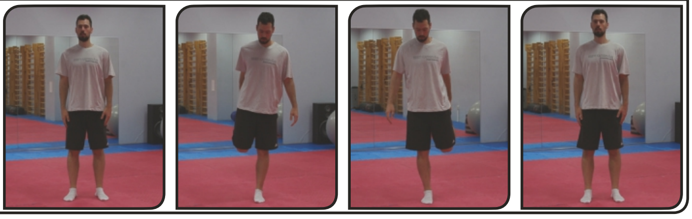

- eccentric squat, 5x

Slow descent into squat. Avoid dynamic knee valgus movement pattern.
Maintain slight extension in the lower back.

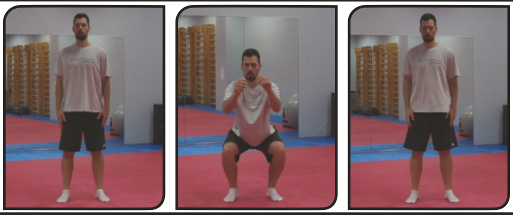

- split squat, 5x bilaterally

Avoid dynamic knee valgus movement pattern.

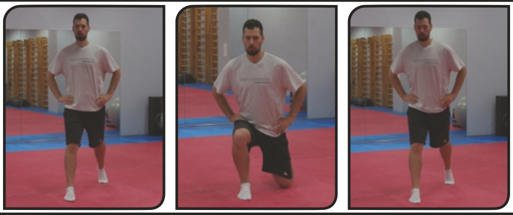

- single-leg deadlift, 5x bilaterally

Avoid dynamic knee valgus movement pattern.
Maintain slight extension in the lower back.

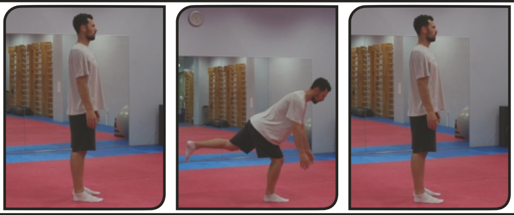

Ankle

- dynamic calf stretch, 5x bilaterally

Controlled, smooth movement within comfortable limits.

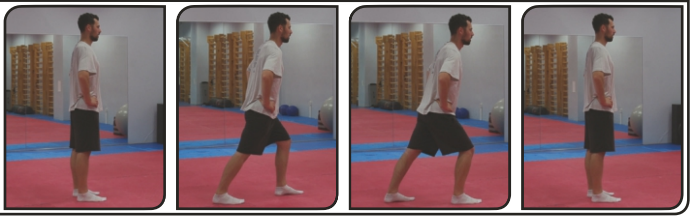

- bodyweight plantar flexion, 10x

Controlled, smooth movement within comfortable limits.

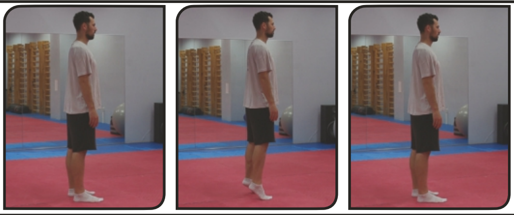

Part 2. Jogging. 5 min

You should choose a pace that would allow you to jog without intense ventilation.

Part 3. Jumping, balance and running exercises with a change of direction. 5 min

- two-leg jump and landing, 5x

Avoid dynamic knee movement pattern.

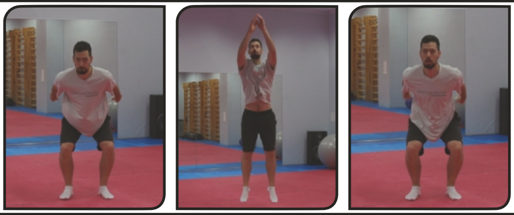

- single-leg landing, 3x each

Low two-leg jump, landing on a single leg.
Avoid dynamic knee movement pattern.

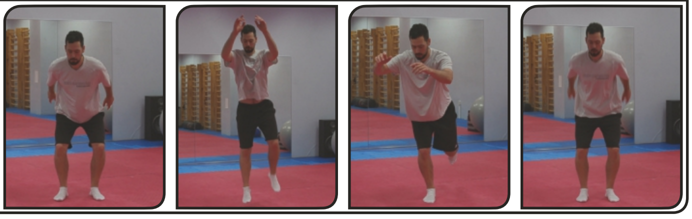

- zig-zag balance run, 5x stopping on each leg

Zig-zag running pattern with movement stoppage and balancing on a single leg before a change of direction. Avoid dynamic knee valgus movement pattern.

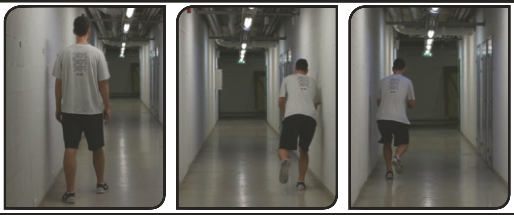

- forward-backward accelerations, 2x change of direction with each leg

Avoid dynamic knee valgus movement pattern.

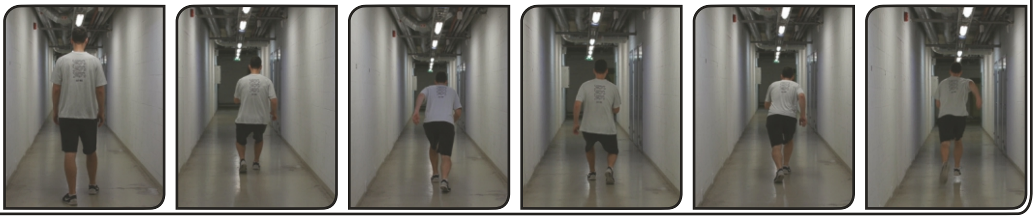

- side shuffle (start from right to left) + forward acceleration, 2x change of direction with each leg

Avoid dynamic knee valgus movement pattern.

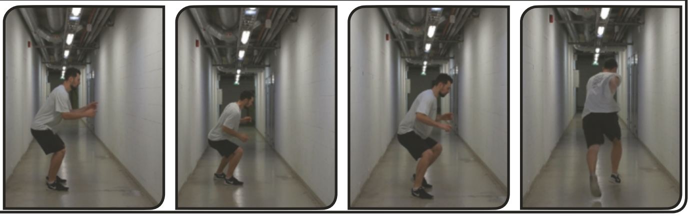

- side shuffle (start from left to right) + forward acceleration, 2x change of direction with each leg

Avoid dynamic knee valgus movement pattern.

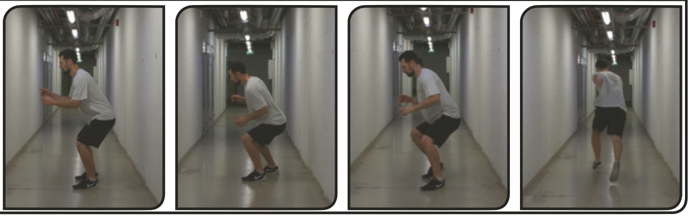

Supplement: Supplementary file 1 [file medicina-59-00356-s001.zip › medicina-2190593-supplementary.pdf]
